# Regulation of cytokine and chemokine expression by histone lysine methyltransferase MLL1 in rheumatoid arthritis synovial fibroblasts

**DOI:** 10.1038/s41598-024-60860-7

**Published:** 2024-05-09

**Authors:** Keita Okamoto, Yasuto Araki, Yoshimi Aizaki, Shinya Tanaka, Yuho Kadono, Toshihide Mimura

**Affiliations:** 1 Department of Rheumatology and Applied Immunology, Faculty of Medicine, Saitama Medical University, 38 Morohongo, Moroyama-chou, Iruma-gun, Saitama, 350-0495 Japan; 2 Department of Orthopaedic Surgery, Faculty of Medicine, Saitama Medical University, 38 Morohongo, Moroyama-chou, Iruma-gun, Saitama, 350-0495 Japan; 3 Department of Orthopedic Surgery, Japan Community Health Care Organization Saitama Northern Medical Center, 1-851, Miyahara-cho, Kita-ku, Saitama-shi, Saitama, 331-8625 Japan

**Keywords:** Rheumatoid arthritis, Synovial fibroblast, Histone lysine methylation, Cytokine, Chemokine, Rheumatology, Rheumatic diseases

## Abstract

Histone lysine methylation is thought to play a role in the pathogenesis of rheumatoid arthritis (RA). We previously reported aberrant expression of the gene encoding mixed-lineage leukemia 1 (MLL1), which catalyzes methylation of histone H3 lysine 4 (H3K4), in RA synovial fibroblasts (SFs). The aim of this study was to elucidate the involvement of MLL1 in the activated phenotype of RASFs. SFs were isolated from synovial tissues obtained from patients with RA or osteoarthritis (OA) during total knee joint replacement. MLL1 mRNA and protein levels were determined after stimulation with tumor necrosis factor α (TNFα). We also examined changes in trimethylation of H3K4 (H3K4me3) levels in the promoters of RA-associated genes (matrix-degrading enzymes, cytokines, and chemokines) and the mRNA levels upon small interfering RNA-mediated depletion of MLL1 in RASFs. We then determined the levels of H3K4me3 and mRNAs following treatment with the WD repeat domain 5 (WDR5)/MLL1 inhibitor MM-102. H3K4me3 levels in the gene promoters were also compared between RASFs and OASFs. After TNFα stimulation, MLL1 mRNA and protein levels were higher in RASFs than OASFs. Silencing of MLL1 significantly reduced H3K4me3 levels in the promoters of several cytokine (interleukin-6 [IL-6], IL-15) and chemokine (C–C motif chemokine ligand 2 [CCL2], CCL5, C-X-C motif chemokine ligand 9 [CXCL9], CXCL10, CXCL11, and C-X3-C motif chemokine ligand 1 [CX3CL1]) genes in RASFs. Correspondingly, the mRNA levels of these genes were significantly decreased. MM-102 significantly reduced the promoter H3K4me3 and mRNA levels of the CCL5, CXCL9, CXCL10, and CXCL11 genes in RASFs. In addition, H3K4me3 levels in the promoters of the IL-6, IL-15, CCL2, CCL5, CXCL9, CXCL10, CXCL11, and CX3CL1 genes were significantly higher in RASFs than OASFs. Our findings suggest that MLL1 regulates the expression of particular cytokines and chemokines in RASFs and is associated with the pathogenesis of RA. These results could lead to new therapies for RA.

## Introduction

Rheumatoid arthritis (RA) is a chronic destructive inflammatory disease that primarily affects the joints^1^. The inflammatory process in RA leads to the activation of synovial fibroblasts (SFs), also called fibroblast-like synoviocytes (FLS), and destruction of the articular cartilage and bone, resulting in joint disability^2^. RASFs produce several matrix metalloproteinase (MMPs), cathepsins, cytokines, and chemokines^3,4^. MMPs, including MMP-1, MMP-3, MMP-9, and MMP-13, and cathepsins, such as Cathepsin K (CTSK) and CTSL, are matrix-degrading enzymes that cause cartilage destruction in RA^5–7^. Cytokines, such as interleukin-6 (IL-6), IL-8, IL-15, and IL-23A, play a critical role in persistent inflammation in RA joints^8–11^. Chemokines, including C–C motif chemokine ligand 2 (CCL2), CCL3, and CCL5; C-X-C motif chemokine ligand 1 (CXCL1), CXCL5, CXCL6, CXCL9, CXCL10, CXCL11, CXCL12, and CXCL13; and C-X3-C motif chemokine ligand 1 (CX3CL1), contribute to RA pathogenesis via the recruitment of immune cells^12–19^. Several cytokines and chemokines also induce a low-grade inflammation in osteoarthritis (OA) joints^20^.

RA is an autoimmune disease characterized by the appearance of autoantibodies, such as rheumatoid factors (RF) and anti-citrullinated protein/peptide antibodies (ACPA)^21,22^. A line of evidence has shown that both genetic and environmental factors are involved in the pathogenesis of RA^23^. However, these factors do not fully explain the pathogenesis of RA. Epigenetic mechanisms are also thought to play a role in the pathogenesis of autoimmune diseases such as RA^24–26^. Epigenetics refers to heritable phenotypic changes that influence gene expression independent of the DNA sequence^27,28^. The mechanisms of such epigenetic changes include DNA methylation, covalent posttranslational modifications of histone tails, expression of non-coding RNAs, and adenosine triphosphate (ATP)-dependent chromatin remodeling. These mechanisms control the change in chromatin structure between the open state (euchromatin) and the closed state (heterochromatin). Euchromatin enhances gene transcription, whereas heterochromatin silences gene transcription. We previously reported that altered histone modifications are associated with the pathogenesis of RA^29–31^. Histone lysine methylation (HKM) is associated with activation of MMP genes in RASFs^5,32^. In addition, we showed that RASFs exhibit aberrant mRNA expression of histone lysine methyltransferases (HKMTs), including myeloid/lymphoid or mixed-lineage leukemia 1 (MLL1, also called lysine methyltransferase 2A [KMT2A])^33^. MLL1 catalyzes the methylation of histone H3 lysine 4 (H3K4), an active histone marker associated with euchromatin^34^. MLL1 regulates the homeobox (HOX) gene expression, which has been implicated in haematopoiesis and embryonic development^35^. Mapping the genomic binding sites of MLL1 also identified approximately 20,000 promoters, suggesting that MLL1 is associated with the regulation of a number of gene transcription in a variety of cells^36^. Although MLL1 is suggested to be involved in the pathogenesis of RA, the mechanisms remain unknown.

In this study, we clarified the role of MLL1 in the pathogenesis of RA by repressing MLL1 expression in RASFs using small interfering RNA (siRNA) experiments. We assessed the levels of trimethylation of H3K4 (H3K4me3) in the genes upon siRNA-mediated depletion of MLL1 in RASFs and identified the genes regulated by MLL1 in RASFs. MLL1 is a part of a complex of proteins associated with Set1 (COMPASS)-like complex that consists of WD repeat domain 5 (WDR5) and other components. WDR5 is required for H3K4 methylation by MLL1. MM-102 is a small-molecule WDR5/MLL1 inhibitor that inhibits the protein–protein interaction between MLL1 and WDR5 and the enzyme activity of MLL1 for H3K4 methylation^37^. We investigated the effect of MM-102 on RA-associated genes in RASFs. Furthermore, H3K4me3 levels in RASFs and OASFs were compared to confirm that HKM is associated with RA pathogenesis.

## Results

### MLL1 is highly expressed in RASFs after tumor necrosis factor α (TNFα) stimulation

**Figure 1:**
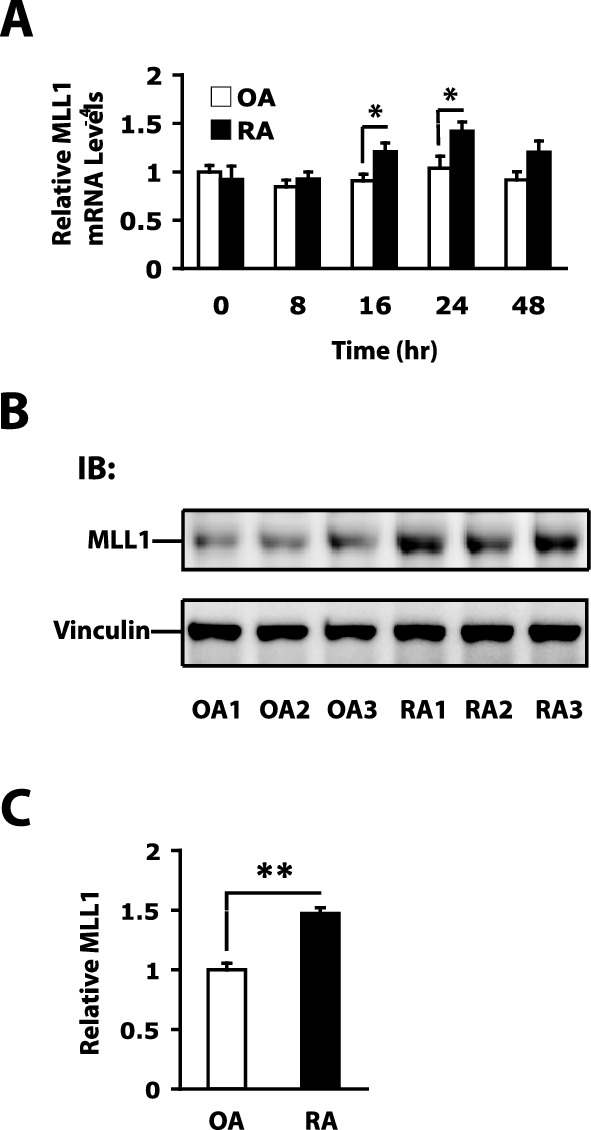
Enhanced expression of mixed-lineage leukemia 1 (MLL1) after tumor necrosis factor α (TNFα) stimulation in rheumatoid arthritis (RA) synovial fibroblasts (SFs) compared to osteoarthritis (OA) SFs. (**A**) MLL1 mRNA levels in OASFs and RASFs at various time points (0, 8, 16, 24, and 48 h) after stimulation with 10 ng/ml TNFα, as determined by quantitative reverse transcriptase (RT) − polymerase chain reaction (PCR). Bars show the mean ± standard error of the mean (SEM) (n = 7 OA patients and 7 RA patients). Values are expressed as the fold-increase versus the value for OASFs at 0 h. (**B**) Representative MLL1 protein expression patterns in SFs from 3 OA patients and 3 RA patients by western blotting analysis. Vinculin was used as the internal control. Original blots are presented in Supplementary Figs. 1A and B. (**C**) The quantified data of MLL1 protein levels are presented as mean ± SEM (n = 7 OA patients and 7 RA patients). The intensity of MLL1 was normalized based on Vinculin. * = *P* < 0.05; ** = *P* < 0.01 by Mann–Whitney *U* test.

Previous studies reported that aberrant HKM dysregulates gene transcription in RASFs, suggesting that HKM is involved in the pathogenesis of RA^30,31^. To determine the mechanism leading to aberrant HKM in RASFs, we investigated the mRNA expression of HKMTs^33^. Aberrant mRNA expression of several HKMTs, including MLL1, which contributes to the generation of H3K4me3 in the promoter and increased gene transcription, was observed in RASFs. Compared with OASFs, MLL1 expression was higher in RASFs at 24 h after TNFα stimulation. To confirm these data, MLL1 mRNA levels were examined in RASFs and OASFs at several time points (0, 8, 16, 24, and 48 h) after stimulation with 10 ng/ml TNFα. MLL1 mRNA levels were significantly higher in RASFs than OASFs at 16 and 24 h after TNFα stimulation (Fig. 1A). This result is consistent with the previous data. Then, MLL1 protein levels were examined by western blotting analysis in RASFs and OASFs at 24 h after TNFα stimulation (Fig. 1B and Supplementary Fig. 1). The MLL1 protein levels were significantly higher in RASFs than OASFs (Fig. 1C). Vinculin was used as the internal control. The results suggest that enhanced MLL1 expression may lead to an increase in H3K4me3 levels in RASFs, possibly resulting in the gene activation associated with the phenotype of RASFs.

### MLL1 depletion decreases H3K4me3 levels in cytokine and chemokine promoters in RASFs

**Figure 2:**
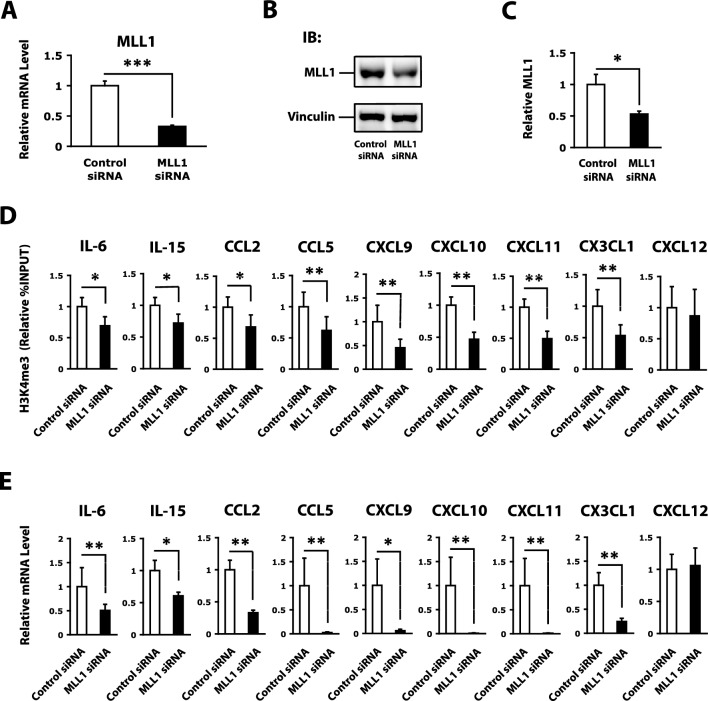
Reduction in levels of trimethylation of histone H3 lysine 4 (H3K4me3) and mRNAs of cytokine and chemokine genes in RASFs upon small interfering RNA (siRNA)-mediated inhibition of MLL1. (**A**) MLL1 mRNA levels in RASFs treated with control siRNA or MLL1 siRNA, as determined by quantitative RT-PCR (n = 12 RA patients). (**B**) Representative MLL1 protein expression patterns in RASFs treated with control siRNA or MLL1 siRNA by western blotting analysis. Vinculin was used as the internal control. Original blots are presented in Supplementary Figs. 2A and B. (**C**) The quantified data of MLL1 protein levels are presented as mean ± SEM (n = 7 RASFs that were treated with control siRNA or MLL1 siRNA). The intensity of MLL1 was normalized based on Vinculin. (**D**) H3K4me3 levels of interleukin (IL)-6, IL-15, C–C motif chemokine ligand (CCL)2, CCL5, C-X-C motif chemokine ligand (CXCL)9, CXCL10, CXCL11, C-X3-C motif chemokine ligand (CX3CL)1, and CXCL12 genes in RASFs treated with control siRNA or MLL1 siRNA, as determined by quantitative chromatin immunoprecipitation (ChIP)-PCR (n = 8 RA patients). (**E**) IL-6, IL-15, CCL2, CCL5, CXCL9, CXCL10, CXCL11, CX3CL1, and CXCL12 mRNA levels in RASFs treated with control siRNA or MLL1 siRNA, as determined by quantitative RT-PCR (n = 12 RA patients). Bars show the mean ± SEM. Values are expressed as the fold-increase versus the value for control siRNA-treated RASFs. * = *P* < 0.05; ** = *P* < 0.01; *** = *P* < 0.001 by Wilcoxon signed-rank test. See Fig. 1 for other definitions.

To identify the genes that are specifically regulated by MLL1 in RASFs, we treated RASFs with MLL1 siRNA and investigated the changes in H3K4me3 levels in the promoters of several genes associated with the pathogenesis of RA, including MMPs, cathepsins, cytokines, and chemokines. MLL1 mRNA levels were significantly repressed upon siRNA-mediated depletion of MLL1 in RASFs (Fig. 2A). MLL1 protein levels were also examined by western blotting analysis (Fig. 2B and Supplementary Fig. 2). The MLL1 protein levels significantly decreased in MLL1 siRNA-treated RASFs (Fig. 2C). Vinculin was used as the internal control. Silencing of MLL1 significantly decreased the H3K4me3 levels of two cytokines (IL-6, IL-15) and six chemokines (CCL2, CCL5, CXCL9, CXCL10, CXCL11, and CX3CL1) in RASFs (Fig. 2D). CXCL12 was shown as an example of negative controls. Therefore, MLL1 is thought to be responsible for the activation of cytokine and chemokine genes associated with the active phenotype of RASFs via H3K4 methylation in the promoters.

### MLL1 depletion represses cytokine and chemokine expression in RASFs

To determine whether an MLL1-induced increase in H3K4me3 levels contributes to the activation of cytokine and chemokine genes in RASFs, changes in cytokine and chemokine gene expression were examined following siRNA-mediated depletion of MLL1. mRNA levels of the IL-6, IL-15, CCL2, CCL5, CXCL9, CXCL10, CXCL11, and CX3CL1 genes were decreased in MLL1 siRNA-treated RASFs (Fig. 2E). CXCL12 was shown as an example of negative controls. These results demonstrate that MLL1 regulates cytokine and chemokine gene activation in RASFs.

### MLL1 inhibition reduces cytokine and chemokine expression via H3K4 methylation in RASFs

**Figure 3:**
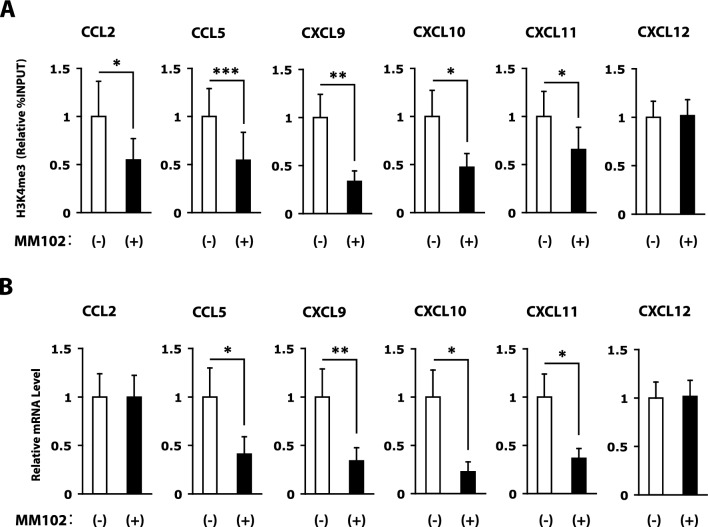
Reduction in H3K4me3 and mRNA levels of cytokine and chemokine genes in RASFs following treatment with the WD repeat domain 5 (WDR5)/MLL1 inhibitor MM-102. (**A**) H3K4me3 levels of the CCL2, CCL5, CXCL9, CXCL10, CXCL11, and CXCL12 genes in RASFs treated with or without the treatment of 300 pM MM-102 for 72 h, as determined by quantitative ChIP-PCR (n = 8 RA patients). (**B**) CCL2, CCL5, CXCL9, CXCL10, CXCL11, and CXCL12 mRNA levels in RASFs treated with or without the treatment of 300 pM MM-102 for 72 h, as determined by quantitative RT-PCR (n = 12 RA patients). Bars show the mean ± SEM. Values are expressed as the fold-increase versus the value for RASFs not treated with MM-102. * = *P* < 0.05; ** = *P* < 0.01; *** = *P* < 0.001 by Wilcoxon signed-rank test. See Figs. 1 and 2 for other definitions.

The WDR5/MLL1 inhibitor MM-102 inhibits the HKMT activity of MLL1. We treated RASFs with 300 pM MM-102 for 72 h to further confirm the effect of MLL1 on the expression of cytokine and chemokine genes in RASFs. MM-102 reduced H3K4me3 levels in the promoters of the CCL2, CCL5, CXCL9, CXCL10, and CXCL11 genes in RASFs (Fig. 3A). MM-102 treatment also decreased CCL5 CXCL9, CXCL10, and CXCL11 mRNA levels in RASFs (Fig. 3B). The CCL2 mRNA level was not affected by MM-102 treatment in RASFs. CXCL12 was shown as an example of negative controls. MM-102 decreased the expression of some of the cytokine and chemokine genes that were repressed upon siRNA-mediated depletion of MLL1. The inhibitory effect of MM-102 on the HKMT activity of MLL1 appeared to be weaker than that of MLL1 siRNA.

### H3K4 methylation is increased in cytokine and chemokine gene promoters in RASFs

**Figure 4:**
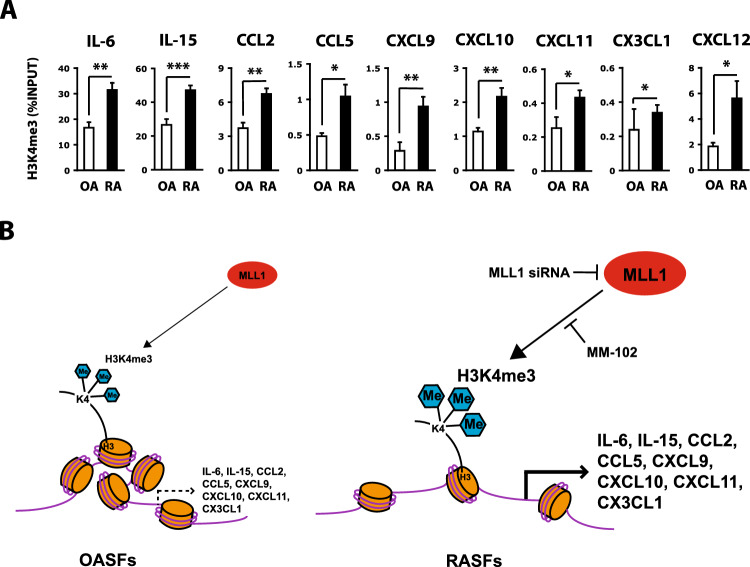
High levels of H3K4me3 that are induced by MLL1 in the promoters of cytokine and chemokine genes in RASFs. (**A**) Levels of H3K4me3 in the IL-6, IL-15, CCL2, CCL5, CXCL9, CXCL10, CXCL11, and CX3CL1 promoters in OASFs and RASFs, as determined by quantitative ChIP-PCR. Bars show the mean ± SEM (n = 8 OA patients and 14 RA patients). H3K4me3 levels are normalized by input DNA and are expressed as %INPUT. * = *P* < 0.05; ** = *P* < 0.01; *** = *P* < 0.001 by Mann–Whitney *U* test. (**B**) A scheme for regulation of cytokine and chemokine gene expression by MLL1-mediated H3K4me3 in OASFs and RASFs. Highly expressed MLL1 leads to cytokine and chemokine gene activation by increasing H3K4me3 levels in RASFs compared with OASFs. See Figs. 1, 2, and 3 for other definitions.

To determine whether H3K4 methylation contributes to the cytokine and chemokine gene expression in RASFs, H3K4me3 levels were compared between RASFs and OASFs. H3K4me3 levels in the cytokine and chemokine promoters were significantly higher in RASFs than OASFs (Fig. 4A). Considering that MLL1 expression is higher in RASFs than OASFs, these results suggest that MLL1 plays a role in the cytokine and chemokine gene activation by this epigenetic change.

## Discussion

The present study demonstrated that MLL1 enhances the expression of distinct cytokines and chemokines, including IL-6, IL-15, CCL2, CCL5, CXCL9, CXCL10, CXCL11, and CX3CL1, via H3K4 methylation in RASFs (Fig. 4B). We also showed that the levels of H3K4me3 in the promoters of these cytokine and chemokine genes are upregulated in RASFs. These results suggest that MLL1 is responsible for the activation of RASFs. We isolated RASFs from synovial tissues obtained from RA patients during total knee joint replacement. As the patients in this study required joint arthroplasty due to RA, we believe the RASFs analyzed in this study to be from RA patients with poor prognosis or destructive arthritis. Regarding the mechanism by which HKM mediates the pathogenesis of RA, a similar result has already been reported as follows. Enhancer of zeste homolog 2 (EZH2), which methylates histone H3K27, was shown to regulate the transcription of Wnt signaling genes in RASFs^38^. Epigenetic regulation is thought to play a role in the pathogenesis of RA.

Cytokine- and chemokine-mediated immune responses have been shown to contribute to the pathogenesis of RA. Previous studies showed that IL-6, IL-15, CCL2, CCL5, CXCL9, CXCL10, CXCL11, and CX3CL1 are highly expressed in RASFs^8,10,12,13,15,16,19^. In this study, we showed high levels of H3K4me3 in these cytokine and chemokine genes in RASFs. Our data thus suggest that these cytokines and chemokines play a pivotal role in the activation of RASFs under epigenetic mechanisms. IL-6, a major pro-inflammatory cytokine with pleiotropic functions^39^, is abundantly produced in RASFs and stimulates the growth and activation of RASFs in an autocrine manner^40^. IL-15 belongs to the 4 α-helix bundle cytokine family and plays an essential role in T helper 17 (Th17) cell activation and proliferation^41,42^. IL-15 produced by RASFs induces IL-17 expression in T cells of RA patients^43^. CCL2, also called monocyte chemoattractant protein-1 (MCP-1), binds to C–C motif chemokine receptor 2 (CCR2) and mediates the migration of effector T cells to the synovium tissue in RA^44,45^. CCL5, also known as regulated on activation, normal T cell expressed and secreted (RANTES), is primarily associated with CCR5^46^. RASFs highly express CCL5, which induces MMP-1 and MMP-13 production^47,48^. CXCL9, also called monokine induced by interferon-γ (MIG), binds to CXC motif chemokine receptor 3 (CXCR3)^49^. RASFs produce CXCL9, which recruits CXCR3 − expressing plasma cells to the synovium^15,47^. CXCL10, also called interferon γ-induced protein 10 (IP-10), is a ligand of CXCR3^50^. CXCL10 is highly secreted by RASFs and enhances RASF invasion in an autocrine manner^16,47,51^. CXCL11, also called interferon-inducible T-cell α chemoattractant (I-TAC), binds to CXCR3 and polarizes CD4^+^ T cells toward the Th2 or IL-10^high^ T regulatory 1 (Tr1) cell phenotype^52–54^. Although CXCL11 may restrain inflammation, the function of CXCL11 in RA remains to be fully elucidated. CX3CL1 is also known as fractalkine and interacts with CX3C motif chemokine receptor 1 (CX3CR1)^55^. RASFs highly express CX3CL1, which induces the migration of monocytes, T cells, and osteoclast precursors into the RA synovium^19^. CXCL12, also known as stromal cell-derived factor 1 (SDF1), binds to CXCR4 and chemotactic for lymphocytes and monocytes but not neutrophils^56^. Although CXCL12 is not thought to be regulated by MLL1, CXCL12 expression levels are positively correlated with RA disease activity^57^.

H3K4me3 levels in the CCL2 promoter and CCL2 mRNA levels were lower in RASFs treated with MLL1 siRNA than RASFs treated with control siRNA (Fig. 2D and E). On the other hand, although H3K4me3 levels in the CCL2 promoter were lower in RASFs treated with MM-102 than RASFs not treated with MM-102 (Fig. 3A), CCL2 mRNA levels were similar between MM-102-treated and non-MM-102-treated RASFs (Fig. 3B). These results seem contradictory. MM-102 suppresses the generation of H3K4me3 by MLL1. In RASFs that were not treated with MM-102, H3K4me3 levels in the CCL2 promoter increased but CCL2 mRNA levels did not increase. Elevated H3K4me3 in the CCL2 gene in RASFs not treated with MM-102 may not have been sufficient to open the chromatin structure enough to increase CCL2 gene transcription. Alternatively, even if the chromatin structure of the CCL2 gene had been open in RASFs not treated with MM-102, there might not have been any transcription factors that would have activated the CCL2 gene transcription. This study demonstrates that MM-102 reduces the expression of several chemokines that play important roles in the pathogenesis of RA. Hence, MLL1 may be a novel and promising target for RA therapy. To determine whether MM-102 would be a useful agent in RA therapy, we are planning a therapeutic study using an animal arthritis model, such as collagen-induced arthritis (CIA).

MLL1 methylates a number of genes in a variety of cells^36^. Although a normal control group is desired in this study, we suspect that there are few cells whose gene expression is not regulated by MLL1. Therefore, OASFs would not be a normal control group in the experiments of MLL1 siRNA and MM-102. However, when RASFs were treated with MLL1 siRNA, the levels of H3K4me3 in cytokine and chemokine genes other than CXCL12 were reduced to the same extent as in OASFs (Figs. 2D and 4A). This indirectly implies that the increase in H3K4me3 in RASFs compared to OASFs may be regulated by RA-specific pathogenic activity of MLL1. The level of H3K4me3 in the CXCL12 gene may be increased by any HKMT other than MLL1 in RASFs.

In conclusion, the results of this study shed light on the role of MLL1 in the epigenetic mechanisms involved in the regulation of H3K4 methylation in distinct cytokine and chemokine genes in RASFs. As cytokines and chemokines play important roles in the activation of RASFs, MLL1 may be a critical regulator in the development of RA. We hope that the results of this study will help facilitate the development of a novel therapy for RA.

## Methods

### Patients and SFs

This study enrolled patients who were diagnosed with RA according to the 1987 American College of Rheumatology (ACR) revised criteria or the 2010 ACR/European League Against Rheumatism (EULAR) classification criteria for the diagnosis of RA^58,59^. Human synovial tissues, which were obtained from RA and OA patients during total knee joint replacement at the Saitama Medical University Hospital, were digested with 1.5 mg/ml collagenase and 0.04% hyaluronidase at 37 °C for 2 h and harvested after 4–8 passages. Seven RASFs and 7 OASFs were stimulated with 10 ng/ml recombinant human TNFα (Peprotech). Twelve RASFs were treated with 10 μM siRNA specifically targeting human MLL1 (Santa Cruz Biotechnology) for 48 h. Twelve RASFs were treated with 300 pM MM-102 (Tocris) for 72 h.

### Quantitative real-time reverse transcription (RT)—polymerase chain reaction (PCR)

Total RNA was isolated from SFs using an RNeasy kit (Qiagen), according to the manufacturer’s instructions. The quantity of total RNA was measured by NanoDrop (Agilent) and 500 ng of total RNA was used to synthesize cDNA using Superscript III (ThermoFisher Scientific), according to the manufacturer’s instructions. In preparation for the RT-PCR analysis, 20-μl aliquots of diluted cDNA, 0.125 μM of each primer, and Power SYBR green PCR master mix (ThermoFisher Scientific) were amplified for 35 cycles using a StepOnePlus Real-Time PCR System (ThermoFisher Scientific), according to the manufacturer’s instructions. The threshold cycle (Ct) of genes was normalized with that of 18S ribosomal RNA (18S rRNA). Gene expression values are shown as 2^−ΔCt^. The sequences of the primers are shown in Supplementary Table 1. All reactions were performed in dupulicate.

### Western blotting analysis

SFs were washed once in PBS, resuspended in lysis buffer (50 mM Tris–HCl, pH 8, 150 mM NaCl, 1% Nonidet P-40, 0.5% sodium deoxycholate, and 0.1% SDS, and protease inhibitors) supplemented with complete protease inhibitor cocktail Tablets (Roche), and incubated on ice for 30 min. Insoluble material was removed by centrifugation at 14,000*g* at 4 °C for 15 min. After heating at 70 °C for 10 min in NuPage LDS sample buffer (ThermoFisher Scientific), 25 μg samples per well were loaded and separated by Tris–Glycine gels (ThermoFisher Scientific). The proteins were transferred onto a polyvinylidene difluoride membrane using an XCell II system (ThermoFisher Scientific). The membrane was blocked with 5% BSA at room temperature for 1 h and incubated with primary antibodies (anti-MLL1 [Merck Millipore] or anti-vinculin [Abcam]) at room temperature for 1 h. Vinculin was used as the internal control. The membrane was subsequently washed three times and incubated with secondary horseradish peroxidase (HRP)-conjugated antibodies (GE Healthcare) at room temperature for 1 h. Then, the membrane was washed three times and developed for 1 min in ECL Western Blotting Detection reagents (GE Healthcare). Signals were detected by chemo-luminescence, captured using an LAS-1000plus luminescent image analyzer (Fujifilm), and quantified by ImageQuant TL (Cytiva), according to the manufacturer’s instructions. The intensity of MLL1 was normalized based on Vinculin.

### Chromatin immunoprecipitation (ChIP)

The native ChIP protocol was described previously^60,61^. SFs (5 × 10^5^ cells) were digested with 2 units of micrococcal nuclease at 37 °C for 10 min. The lysates were dialyzed against RIPA buffer at 4 °C for 2 h and then incubated with Dynabeads protein G (ThermoFisher Scientific) and anti-H3K4me3 (Merck Millipore) at 4 °C overnight. After incubation with 0.2 mg/ml RNase A and 1 mg/ml proteinase K at 65 °C overnight, the immunoprecipitated DNA was purified and used for real-time PCR analysis. The amount of immunoprecipitated DNA was normalized to that of input DNA. The sequences of the primers are shown in Supplementary Table 2. All reactions were performed in dupulicate.

### Statistics

Data were expressed as the mean ± standard error of the mean (SEM). Differences between unpaired or paired groups were evaluated using the Mann–Whitney *U* test or the Wilcoxon signed-rank test, respectively. In all analyses, a *P* value less than 0.05 was considered statistically significant. All analyses were conducted using JMP 6.0 Software (SAS Institute).

### Ethical approval

This study was approved by the Ethics Committee of Saitama Medical University (approval number: 620-VI). The study was performed in accordance with the ethical standards in the Declaration of Helsinki and the relevant guidelines and regulations. Written informed consent was obtained from every patient and all samples were rendered anonymous. Anonymity and confidentiality were ensured.

### Supplementary Information

Supplementary Figure 1.

Supplementary Figure 2.

Supplementary Table 1.

Supplementary Table 2.

Supplementary Legends.

## Data Availability

The data generated and/or analysed during the current study are available from the corresponding author on reasonable request.

## References

[1] Smolen JS, Aletaha D, Barton A, Burmester GR, Emery P, Firestein GS, Kavanaugh A, McInnes IB, Solomon DH, Strand V (2018). Rheumatoid arthritis. Nat. Rev. Dis. Primers.

[2] Bartok B, Firestein GS (2010). Fibroblast-like synoviocytes: Key effector cells in rheumatoid arthritis. Immunol. Rev..

[3] Noss EH, Brenner MB (2008). The role and therapeutic implications of fibroblast-like synoviocytes in inflammation and cartilage erosion in rheumatoid arthritis. Immunol. Rev..

[4] Elemam NM, Hannawi S, Maghazachi AA (2020). Role of chemokines and chemokine receptors in rheumatoid arthritis. ImmunoTargets Ther..

[5] Araki Y, Mimura T (2017). Matrix Metalloproteinase gene activation resulting from disordred epigenetic mechanisms in rheumatoid arthritis. Int. J. Mol. Sci..

[6] Hou WS, Li W, Keyszer G, Weber E, Levy R, Klein MJ, Gravallese EM, Goldring SR, Bromme D (2002). Comparison of cathepsins K and S expression within the rheumatoid and osteoarthritic synovium. Arthritis Rheum..

[7] Schedel J, Seemayer CA, Pap T, Neidhart M, Kuchen S, Michel BA, Gay RE, Muller-Ladner U, Gay S, Zacharias W (2004). Targeting cathepsin L (CL) by specific ribozymes decreases CL protein synthesis and cartilage destruction in rheumatoid arthritis. Gene Ther..

[8] Hirano T, Matsuda T, Turner M, Miyasaka N, Buchan G, Tang B, Sato K, Shimizu M, Maini R, Feldmann M (1988). Excessive production of interleukin 6/B cell stimulatory factor-2 in rheumatoid arthritis. Eur. J. Immunol..

[9] Xu L, Feng X, Tan W, Gu W, Guo D, Zhang M, Wang F (2013). IL-29 enhances Toll-like receptor-mediated IL-6 and IL-8 production by the synovial fibroblasts from rheumatoid arthritis patients. Arthritis Res. Ther..

[10] Benito-Miguel M, Garcia-Carmona Y, Balsa A, Bautista-Caro MB, Arroyo-Villa I, Cobo-Ibanez T, Bonilla-Hernan MG, Perez de Ayala C, Sanchez-Mateos P, Martin-Mola E (2012). IL-15 expression on RA synovial fibroblasts promotes B cell survival. PloS one.

[11] Goldberg M, Nadiv O, Luknar-Gabor N, Agar G, Beer Y, Katz Y (2009). Synergism between tumor necrosis factor alpha and interleukin-17 to induce IL-23 p19 expression in fibroblast-like synoviocytes. Mol. Immunol..

[12] Eisinger K, Bauer S, Schaffler A, Walter R, Neumann E, Buechler C, Muller-Ladner U, Frommer KW (2012). Chemerin induces CCL2 and TLR4 in synovial fibroblasts of patients with rheumatoid arthritis and osteoarthritis. Exp. Mol. Pathol..

[13] Clanchy FIL, Williams RO (2018). Ibudilast inhibits chemokine expression in rheumatoid synovial fibroblasts and exhibits immunomodulatory activity in experimental arthritis. Arthritis Rheumatol..

[14] Sato H, Muraoka S, Kusunoki N, Masuoka S, Yamada S, Ogasawara H, Imai T, Akasaka Y, Tochigi N, Takahashi H (2017). Resistin upregulates chemokine production by fibroblast-like synoviocytes from patients with rheumatoid arthritis. Arthritis Res. Ther..

[15] Tsubaki T, Takegawa S, Hanamoto H, Arita N, Kamogawa J, Yamamoto H, Takubo N, Nakata S, Yamada K, Yamamoto S (2005). Accumulation of plasma cells expressing CXCR3 in the synovial sublining regions of early rheumatoid arthritis in association with production of Mig/CXCL9 by synovial fibroblasts. Clin. Exp. Immunol..

[16] Ueno A, Yamamura M, Iwahashi M, Okamoto A, Aita T, Ogawa N, Makino H (2005). The production of CXCR3-agonistic chemokines by synovial fibroblasts from patients with rheumatoid arthritis. Rheumatol. Int..

[17] Karouzakis E, Rengel Y, Jungel A, Kolling C, Gay RE, Michel BA, Tak PP, Gay S, Neidhart M, Ospelt C (2011). DNA methylation regulates the expression of CXCL12 in rheumatoid arthritis synovial fibroblasts. Genes Immun..

[18] Timmer TC, Baltus B, Vondenhoff M, Huizinga TW, Tak PP, Verweij CL, Mebius RE, van der Pouw Kraan TC (2007). Inflammation and ectopic lymphoid structures in rheumatoid arthritis synovial tissues dissected by genomics technology: Identification of the interleukin-7 signaling pathway in tissues with lymphoid neogenesis. Arthritis Rheumatism.

[19] Nanki T, Imai T, Kawai S (2017). Fractalkine/CX3CL1 in rheumatoid arthritis. Modern Rheumatol..

[20] Molnar V, Matišić V, Kodvanj I, Bjelica R, Jeleč Ž, Hudetz D, Rod E, Čukelj F, Vrdoljak T, Vidović D (2021). Cytokines and chemokines involved in osteoarthritis pathogenesis. Int. J. Mol. Sci..

[21] Tan EM, Smolen JS (2016). Historical observations contributing insights on etiopathogenesis of rheumatoid arthritis and role of rheumatoid factor. J. Exp. Med..

[22] Sakkas LI, Bogdanos DP, Katsiari C, Platsoucas CD (2014). Anti-citrullinated peptides as autoantigens in rheumatoid arthritis-relevance to treatment. Autoimmun. Rev..

[23] Costenbader KH, Gay S, Alarcon-Riquelme ME, Iaccarino L, Doria A (2012). Genes, epigenetic regulation and environmental factors: Which is the most relevant in developing autoimmune diseases?. Autoimmun. Rev..

[24] Klein K, Ospelt C, Gay S (2012). Epigenetic contributions in the development of rheumatoid arthritis. Arthritis Res. Ther..

[25] Araki Y, Mimura T (2024). Epigenetic dysregulation in the pathogenesis of systemic lupus erythematosus. Int. J. Mol. Sci..

[26] Araki Y, Mimura T (2024). Epigenetic basis of autoimmune disorders in humans. Epigenetics in Human Disease.

[27] Berger SL, Kouzarides T, Shiekhattar R, Shilatifard A (2009). An operational definition of epigenetics. Genes Dev..

[28] Weng NP, Araki Y, Subedi K (2012). The molecular basis of the memory T cell response: Differential gene expression and its epigenetic regulation. Nat. Rev. Immunol..

[29] Wada TT, Araki Y, Sato K, Aizaki Y, Yokota K, Kim YT, Oda H, Kurokawa R, Mimura T (2014). Aberrant histone acetylation contributes to elevated interleukin-6 production in rheumatoid arthritis synovial fibroblasts. Biochem. Biophys. Res. Commun..

[30] Araki Y, Mimura T (2016). The mechanisms underlying chronic inflammation in rheumatoid arthritis from the perspective of the epigenetic landscape. J. Immunol. Res..

[31] Araki Y, Mimura T (2017). The histone modification code in the pathogenesis of autoimmune diseases. Mediators Inflamm..

[32] Araki Y, Tsuzuki Wada T, Aizaki Y, Sato K, Yokota K, Fujimoto K, Kim YT, Oda H, Kurokawa R, Mimura T (2016). Histone methylation and STAT-3 differentially regulate interleukin-6-induced matrix metalloproteinase gene activation in rheumatoid arthritis synovial fibroblasts. Arthritis Rheumatol..

[33] Araki Y, Aizaki Y, Sato K, Oda H, Kurokawa R, Mimura T (2018). Altered gene expression profiles of histone lysine methyltransferases and demethylases in rheumatoid arthritis synovial fibroblasts. Clin. Exp. Rheumatol..

[34] Shilatifard A (2012). The COMPASS family of histone H3K4 methylases: Mechanisms of regulation in development and disease pathogenesis. Ann. Rev. Biochem..

[35] Yu BD, Hess JL, Horning SE, Brown GA, Korsmeyer SJ (1995). Altered Hox expression and segmental identity in Mll-mutant mice. Nature.

[36] Scacheri PC, Davis S, Odom DT, Crawford GE, Perkins S, Halawi MJ, Agarwal SK, Marx SJ, Spiegel AM, Meltzer PS (2006). Genome-wide analysis of menin binding provides insights into MEN1 tumorigenesis. PLoS Genet..

[37] Karatas H, Townsend EC, Cao F, Chen Y, Bernard D, Liu L, Lei M, Dou Y, Wang S (2013). High-affinity, small-molecule peptidomimetic inhibitors of MLL1/WDR5 protein-protein interaction. J. Am. Chem. Soc..

[38] Trenkmann M, Brock M, Gay RE, Kolling C, Speich R, Michel BA, Gay S, Huber LC (2011). Expression and function of EZH2 in synovial fibroblasts: Epigenetic repression of the Wnt inhibitor SFRP1 in rheumatoid arthritis. Ann. Rheumatic Dis..

[39] Kishimoto T (2005). Interleukin-6: From basic science to medicine–40 years in immunology. Ann. Rev. Immunol..

[40] Okamoto H, Yamamura M, Morita Y, Harada S, Makino H, Ota Z (1997). The synovial expression and serum levels of interleukin-6, interleukin-11, leukemia inhibitory factor, and oncostatin M in rheumatoid arthritis. Arthritis Rheumatism.

[41] Tagaya Y, Bamford RN, DeFilippis AP, Waldmann TA (1996). IL-15: A pleiotropic cytokine with diverse receptor/signaling pathways whose expression is controlled at multiple levels. Immunity.

[42] Yoshihara K, Yamada H, Hori A, Yajima T, Kubo C, Yoshikai Y (2007). IL-15 exacerbates collagen-induced arthritis with an enhanced CD4+ T cell response to produce IL-17. Eur. J. Immunol..

[43] Miranda-Carús ME, Balsa A, Benito-Miguel M, de Pérez Ayala C, Martín-Mola E (2004). IL-15 and the initiation of cell contact-dependent synovial fibroblast-T lymphocyte cross-talk in rheumatoid arthritis: Effect of methotrexate. J. Immunol..

[44] Zhu S, Liu M, Bennett S, Wang Z, Pfleger KDG, Xu J (2021). The molecular structure and role of CCL2 (MCP-1) and C-C chemokine receptor CCR2 in skeletal biology and diseases. J. Cell. Physiol..

[45] Moadab F, Khorramdelazad H, Abbasifard M (2021). Role of CCL2/CCR2 axis in the immunopathogenesis of rheumatoid arthritis: Latest evidence and therapeutic approaches. Life Sci..

[46] Zeng Z, Lan T, Wei Y, Wei X (2022). CCL5/CCR5 axis in human diseases and related treatments. Genes Dis..

[47] Yu X, Song Z, Rao L, Tu Q, Zhou J, Yin Y, Chen D (2020). Synergistic induction of CCL5, CXCL9 and CXCL10 by IFN-γ and NLRs ligands on human fibroblast-like synoviocytes-A potential immunopathological mechanism for joint inflammation in rheumatoid arthritis. Int. Immunopharmacol..

[48] Agere SA, Akhtar N, Watson JM, Ahmed S (2017). RANTES/CCL5 induces collagen degradation by activating MMP-1 and MMP-13 expression in human rheumatoid arthritis synovial fibroblasts. Front. Immunol..

[49] Paparo SR (2019). Rheumatoid arthritis and the Th1 chemokine MIG. La Clinica terapeutica.

[50] Antonelli A, Ferrari SM, Giuggioli D, Ferrannini E, Ferri C, Fallahi P (2014). Chemokine (C-X-C motif) ligand (CXCL)10 in autoimmune diseases. Autoimmun. Rev..

[51] Laragione T, Brenner M, Sherry B, Gulko PS (2011). CXCL10 and its receptor CXCR3 regulate synovial fibroblast invasion in rheumatoid arthritis. Arthritis Rheumatism.

[52] Singh AK, Arya RK, Trivedi AK, Sanyal S, Baral R, Dormond O, Briscoe DM, Datta D (2013). Chemokine receptor trio: CXCR3, CXCR4 and CXCR7 crosstalk via CXCL11 and CXCL12. Cytokine Growth Factor Rev..

[53] Karin N, Wildbaum G, Thelen M (2016). Biased signaling pathways via CXCR3 control the development and function of CD4+ T cell subsets. J. Leukocyte Biol..

[54] Zohar Y, Wildbaum G, Novak R, Salzman AL, Thelen M, Alon R, Barsheshet Y, Karp CL, Karin N (2014). CXCL11-dependent induction of FOXP3-negative regulatory T cells suppresses autoimmune encephalomyelitis. J. Clin. Investig..

[55] D'Haese JG, Friess H, Ceyhan GO (2012). Therapeutic potential of the chemokine-receptor duo fractalkine/CX3CR1: An update. Expert Opin. Therap Targets.

[56] Bleul CC, Fuhlbrigge RC, Casasnovas JM, Aiuti A, Springer TA (1996). A highly efficacious lymphocyte chemoattractant, stromal cell-derived factor 1 (SDF-1). J. Exp. Med..

[57] Peng L, Zhu N, Mao J, Huang L, Yang Y, Zhou Z, Wang L, Wu B (2020). Expression levels of CXCR4 and CXCL12 in patients with rheumatoid arthritis and its correlation with disease activity. Exp. Therapeutic Med..

[58] Arnett FC, Edworthy SM, Bloch DA, McShane DJ, Fries JF, Cooper NS, Healey LA, Kaplan SR, Liang MH, Luthra HS (1988). The American Rheumatism Association 1987 revised criteria for the classification of rheumatoid arthritis. Arthritis Rheumatism.

[59] Aletaha D, Neogi T, Silman AJ, Funovits J, Felson DT, Bingham CO, Birnbaum NS, Burmester GR, Bykerk VP, Cohen MD (2010). 2010 Rheumatoid arthritis classification criteria: An American College of Rheumatology/European League Against Rheumatism collaborative initiative. Arthritis Rheumatism.

[60] Araki Y, Fann M, Wersto R, Weng NP (2008). Histone acetylation facilitates rapid and robust memory CD8 T cell response through differential expression of effector molecules (eomesodermin and its targets: Perforin and granzyme B). J. Immunol..

[61] Araki Y, Wang Z, Zang C, Wood WH, Schones D, Cui K, Roh TY, Lhotsky B, Wersto RP, Peng W (2009). Genome-wide analysis of histone methylation reveals chromatin state-based regulation of gene transcription and function of memory CD8+ T cells. Immunity.

