# Supplementary material for: Regulation of cytokine and chemokine expression by histone lysine methyltransferase MLL1 in rheumatoid arthritis synovial fibroblasts
Source: Sci Rep. 2024 May 9;14:10610. doi: 10.1038/s41598-024-60860-7 (PMC11078978; doi:10.1038/s41598-024-60860-7)
Supplement: Supplementary file 3 — Supplementary Table 1. [file 41598_2024_60860_MOESM3_ESM.doc]

**Supplementary Table 1.** List of primer pairs used for quantitative RT-PCR

gene Primer sequence

*MMP-1* Forward: 5'-CCCAGGTATTGGAGGGGATG-3'

Reverse: 5'-CAGTAGAATGGGAGAGTCCAAGAGA-3'

*MMP-3* Forward: 5'-CAGCACTCTGAGGGGAGAAA-3'

Reverse: 5'-ACGCCTGAAGGAAGAGATGG-3'

*MMP-9* Forward: 5'-ATGCCTGCAACGTGAACATC-3'

Reverse: 5'-ATCGCCAGTACTTCCCATCCT-3'

*MMP-13* Forward: 5'-ACCCCAACCCTAAACATCCAA-3'

Reverse: 5'-AAAACAGCTCCGCATCAACC-3'

*CTSK*  Forward: 5'-GCCTGACCTCCTTCCAGTTT-3'

Reverse: 5'-TCCAGTGCTTGTTTCCCTTC-3'

*CTSL*  Forward: 5'-TGAGGCAACAGAAGAATC-3'

Reverse: 5'-CTCCTGCTTAGGGATGTC-3'

*IL-6*  Forward: 5'-GCCAGAGCTGTGCAGATGAGT-3'

Reverse: 5'-TGGGTCAGGGGTGGTTATTG-3'

*IL-8*  Forward: 5'-ACCTTTCCACCCCAAATTTATC-3'

Reverse: 5'-CCTCTGCACCCAGTTTTCCT-3'

*IL-15*  Forward: 5'-GCAGGGCTTCCTAAAACAG-3'

Reverse: 5'-GCAACTGGGGTGAACATC-3'

*IL-23A*  Forward: 5'-AAGAGAAGAGGGAGATGAAGAGAC-3'

Reverse: 5'-CAAGCAGAACTGACTGTTGTCC-3'

*CCL2*  Forward: 5'-AGCAGCCACCTTCATTCC-3'

Reverse: 5'-GCTTCTTTGGGACACTTGC-3'

*CCL3*  Forward: 5'-GCATCACTTGCTGCTGACAC-3'

Reverse: 5'-CTGGCTGCTCGTCTCAAAG-3'

*CCL5*  Forward: 5'-CAAGGAGTATTTCTACACCAGTGGC-3'

Reverse: 5'-CCCGAACCCATTTCTTCTCTG-3'

*CXCL1* Forward: 5'-CACCCCAAGAACATCCAAAG-3'

Reverse: 5'-TTGAGTGTGGCTATGACTTCG-3'

*CXCL5* Forward: 5'-GCTGCGTTGCGTTTGTTTACAG-3'

Reverse: 5'-TTCTTCAGGGAGGCTACCACTTCC-3'

*CXCL6* Forward: 5'-CGCTGAGAGTAAACCCCAAAAC-3'

Reverse: 5'-AGGCTACCACTTCCACCTTG-3'

*CXCL9* Forward: 5'-GCAAGGAACCCCAGTAGTGAG-3'

Reverse: 5'-TTCAAGGATTGTAGGTGGATAGTC-3'

*CXCL10* Forward: 5'-AGGGTGAGAAGAGATGTC-3'

Reverse: 5'-TTTTAGACCTTTCCTTGC-3'

*CXCL11* Forward: 5'-GTTGTTCAAGGCTTCCCC-3'

Reverse: 5'-CATTATGGAGGCTTTCTC-3'

*CXCL12* Forward: 5'-TCTCAACACTCCAAACTGTG-3'

Reverse: 5'-ACTCCTGAATCCACTTTAGC-3'

*CXCL13* Forward: 5'-CCAAGGTGTTCTGGAGGTC-3'

Reverse: 5'-GGACAACCATTCCCACGG-3'

*CX3CL1* Forward: 5'-GCCACCTTCTGCCATCTGAC-3'

Reverse: 5'-TGCCTGGTTCTGTTGATAGTG-3'

*18S rRNA* Forward: 5'-GTAACCCGTTGAACCCCATT-3'

Reverse: 5'-CCATCCAATCGGTAGTAGCG-3'
