# Supplementary material for: Regulation of cytokine and chemokine expression by histone lysine methyltransferase MLL1 in rheumatoid arthritis synovial fibroblasts
Source: Sci Rep. 2024 May 9;14:10610. doi: 10.1038/s41598-024-60860-7 (PMC11078978; doi:10.1038/s41598-024-60860-7)
Supplement: Supplementary file 4 — Supplementary Table 2. [file 41598_2024_60860_MOESM4_ESM.doc]

**Supplementary Table 2.** List of primer pairs used for quantitative ChIP-PCR

gene Primer sequence

*MMP-1* Forward: 5'-GGAGTCACCATTTCTAATGATTGC-3'

Reverse: 5'-GAGGCTGTCTGACTCATGCTTT-3'

*MMP-3* Forward: 5'-GCTGCGGGTGATCCAAA-3'

Reverse: 5'-TGTCTTGCCTGCCTCCTTGT-3'

*MMP-9* Forward: 5'-AGCACTTGCCTGTCAAGGAG-3'

Reverse: 5'-ATGGTGAGGGCAGAGGTGT-3'

*MMP-13* Forward: 5'-GTGGAAACCTATCCATAAGTGATGA-3'

Reverse: 5'-ATGCCTGGGGACTGTTGTCT-3'

*CTSK*  Forward: 5'-CCGATCACTGGAGCTGACTT-3'

Reverse: 5'-CACCATCAGGGGTGCTAGAT-3'

*CTSL*  Forward: 5'-TCAACTGCTTTTCTCCTGTCAACC-3'

Reverse: 5'-CCTTTCCAGACTCCTCCATTCAG-3'

*IL-6*  Forward: 5'-AATGTGGGATTTTCCCATGA-3'

Reverse: 5'-GCCTCAGACATCTCCAGTCC-3'

*IL-8*  Forward: 5'-CCCCCTAAGAGCAGTAACAGTTCC-3'

Reverse: 5'-TGGTGAAGATAAGCCAGCCAATC-3'

*IL-15*  Forward: 5'-ACACCTGGGGCAGTCAGTCATC-3'

Reverse: 5'-GGGAGCATAGGCGAAGACAAAC-3'

*IL-23A*  Forward: 5'-GCTTCCCAGTTCTCCAAGTTCC-3'

Reverse: 5'-TCCTACCTGATGCCCCCTATTAG-3'

*CCL2*  Forward: 5'-CATCTGTGGTCAGTCTGGGC-3'

Reverse: 5'-TAGGAAAGGGAAGCAGGGGGTCAGG-3'

*CCL3*  Forward: 5'-TCCACAGCATCAGCCCATCAAC-3'

Reverse: 5'-ATGAACTCTCCAGCCCCATTCC-3'

*CCL5*  Forward: 5'-TGAGGGAGAGACAGAGACTCG-3'

Reverse: 5'-CCAAAATAGCAACCAAGCATTG-3'

*CXCL1* Forward: 5'-CCAGGGGATTTTGAAAGTTCCAAC-3'

Reverse: 5'-TGGTGGCAACAGATTATCAGTGTC-3'

*CXCL5* Forward: 5'-TCCCCCAACCTCTTCTTTCCAC-3'

Reverse: 5'-TCTACACTCATCTCTCCCCACTGAC-3'

*CXCL6* Forward: 5'-GCCATTGGAGAGGAGGAGCATC-3'

Reverse: 5'-AGCAGCGATTCAGCAGGGTC-3'

*CXCL9* Forward: 5'-GTGGGTTGTCTGTTTACTCTGC-3'

Reverse: 5'-GGTTAGAATAAGGGGTCAGTTGC-3'

*CXCL10* Forward: 5'-CAAAAAAAGAGGAGCAGAGGG-3'

Reverse: 5'-GCATTACAGTTGACTTAGCAAAACC-3'

*CXCL11* Forward: 5'-ATGTGAAGGATGAAGGGTGGG-3'

Reverse: 5'-ATTGTCGGACTACTTTGGGCAGCG-3'

*CXCL12* Forward: 5'-ATTGAATCTCCCGTCCCACTCC-3'

Reverse: 5'-GGCTCTCAGTAAAAGCGAATGTAGC-3'

*CXCL13* Forward: 5'-AAGAGACATTCATTTGCTGTGC-3'

Reverse: 5'-TGACATTGGGGACTCCGTCG-3'

*CX3CL1* Forward: 5'-CACTCTGATGCTGTGGATAACCTG-3'

Reverse: 5'-AGGGTCTGCTGAAAGAAGGATTG-3'
