# Supplementary material for: Regulation of cytokine and chemokine expression by histone lysine methyltransferase MLL1 in rheumatoid arthritis synovial fibroblasts
Source: Sci Rep. 2024 May 9;14:10610. doi: 10.1038/s41598-024-60860-7 (PMC11078978; doi:10.1038/s41598-024-60860-7)
Supplement: Supplementary file 5 — Supplementary Legends. [file 41598_2024_60860_MOESM5_ESM.doc]

**Figure legends**

**Supplementary Figure 1.** Enhanced expression of mixed-lineage leukemia 1 (MLL1) after tumor necrosis factor α (TNFα) stimulation in rheumatoid arthritis (RA) synovial fibroblasts (SFs) compared to osteoarthritis (OA) SFs. **A,** Western full-length blots of MLL1 protein (300 kDa) expression patterns in SFs from 3 OA patients and 3 RA patients. **B,** Western full-length blots of Vinculin protein (124 kDa) expression patterns in SFs from 3 OA patients and 3 RA patients. These are original unprocessed images including membrane edges.

**Supplementary Figure 2.** Reduction in levels of MLL1 protein in RASFs upon small interfering RNA (siRNA)-mediated inhibition of MLL1. **A,** Western full-length blots of MLL1 protein (300 kDa) expression patterns in a RASF treated with control siRNA or MLL1 siRNA. **B,** Western full-length blots of vinculin protein (124 kDa) expression patterns in a RASF treated with control siRNA or MLL1 siRNA. These are original unprocessed images including membrane edges. See Supplementary Figure 1 for other definitions.
